# Early prediction of response to neoadjuvant chemotherapy in patients with breast cancer using diffusion-weighted imaging and gray-scale ultrasonography

**DOI:** 10.3892/or.2014.3025

**Published:** 2014-02-18

**Authors:** HITOMI IWASA, KEI KUBOTA, NORIHIKO HAMADA, MUNENOBU NOGAMI, AKIHITO NISHIOKA

**Affiliations:** Department of Radiology, Kochi Medical School, Kochi University, Kohasu, Oko-cho, Nankoku, Kochi 783-8505, Japan

**Keywords:** breast, cancer, chemotherapy, diffusion-weighted imaging, prediction, response rate, ultrasonography

## Abstract

Neoadjuvant chemotherapy (NACT) is a widely accepted therapeutic option for patients with breast cancer. Although NACT produces good results for breast cancer patients, it has the potential to delay effective treatment in patients with chemotherapy-resistant breast cancer. The purpose of the present study was to evaluate the utility of the pretreatment apparent diffusion coefficient (ADC), which is calculated from diffusion-weighted imaging (DWI), the change in ADC after first administration of NACT, and the change in tumor greatest diameter on ultrasonography in the early prediction of the tumor response to NACT. The response rate of breast tumors to NACT was calculated by the greatest diameter measured by contrast-enhanced MRI obtained before and after NACT. Only the change in ADC was significantly correlated with the response rate. The area under the curve of the change in ADC was sufficiently high (0.90, 95% confidence interval, 0.760–1.040) to discriminate between responders and non-responders. Calculation of the ADC from DWI-MRI was found to be useful for predicting breast tumor response to NACT. Further studies are required to investigate the benefit of changing systemic therapy for breast cancer based on the prediction of the response to NACT by DWI-MRI.

## Introduction

Neoadjuvant systemic chemotherapy (NACT) is the accepted approach for women with locally advanced breast cancer and is an option for women with operable breast cancer, particularly when mastectomy rather than conservative surgery is indicated and the patient desires an attempt at breast conservation (1–4). The primary established clinical benefit for NACT compared with postoperative or adjuvant therapy is in the downstaging of large tumors to improve surgical options. NACT has been shown to result in significantly increased rates of breast-conserving surgery without adversely affecting the overall and disease-free survival rates compared to adjuvant chemotherapy (5,6). However, NACT may have potential disadvantages by delaying local therapy in patients whose tumors turn out to be resistant to the treatment. In fact, some breast cancers do not respond to NACT (7,8).

Diffusion-weighted imaging (DWI) was originally implemented to discover acute cerebral infarction, but it has been increasingly used for the evaluation of extracranial sites such as the abdomen, pelvis (9–11) and breast (12–18). The apparent diffusion coefficient (ADC) is calculated from DWI and correlates with water diffusion without any need for injected contrast material. Although it was found that the mean percentage ADC increase was higher in responders than in non-responders after all cycles of NACT in patients with breast cancer (13–17), measuring the response to anticancer agents after final NACT administration is too late. By that time, a large quantity of anticancer-agent has already been administered to the patient, and too much time has passed from the time that the breast cancer was discovered. A way of switching from an ineffective anticancer regimen to another method of treatment at an early point is required. A number of studies have reported that the original ADC value before the start of NACT is useful for predicting tumor response to anticancer agents (15,17,18). After the first and second cycle time points of NACT, an increase in the mean ADC was noted sooner than a reduction in the tumor diameter (12). The purpose of the present study was to compare the usefulness of the pretreatment ADC, the change in ADC after the first cycle of NACT, and the change in tumor greatest diameter measured by gray-scale ultrasonography for the early prediction of tumor response to NACT.

## Materials and methods

### Patients

Prospective subjects were 24 consecutive female patients with 24 breast cancers diagnosed between March 2009 and October 2010 according to characteristic imaging findings and positive results on core needle biopsy. Each patient was fully informed about the purposes and potential risks and benefits of the study and they provided written, informed consent prior to enrolment. The present study was performed in accordance with the recommendations of the Declaration of Helsinki. Patient characteristics are shown in Table I. A systemic epirubicin/cyclophosphamide (EC) regimen was administered four times as NACT. The dose with each administration of EC chemotherapy was 90 mg/m^2^ of epirubicin and 600 mg/m^2^ of cyclophosphamide injected every 3 weeks. NACT did not result in adverse events severe enough to warrant withdrawal of therapy after appropriate supportive therapy was provided (such as anti-allergic agents and anti-emetic drugs).

### MRI study (contrast-enhanced MRI)

**Figure 1:**
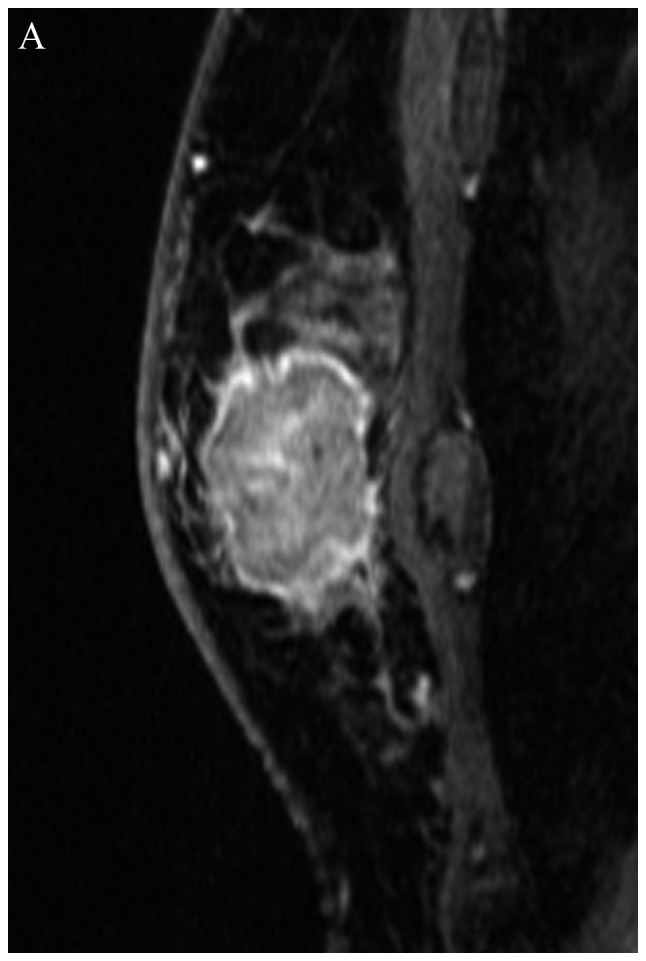

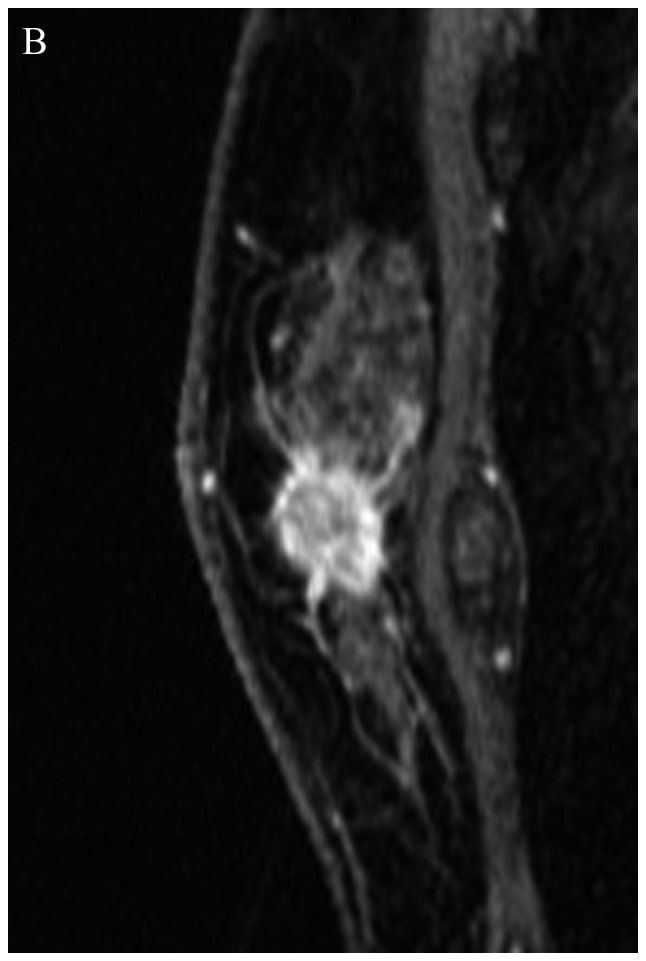

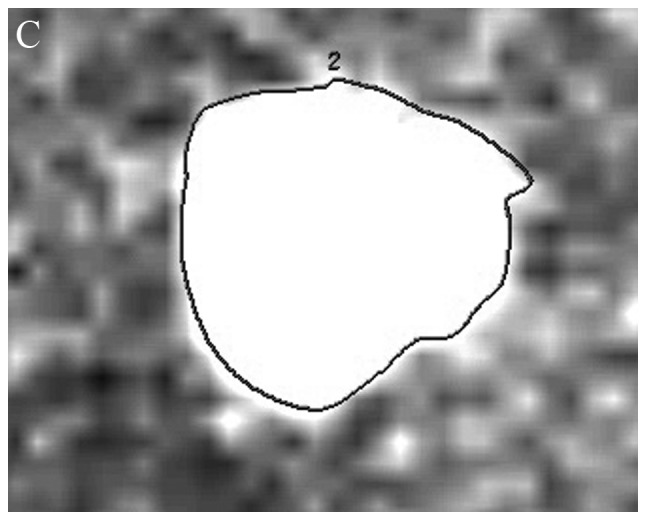

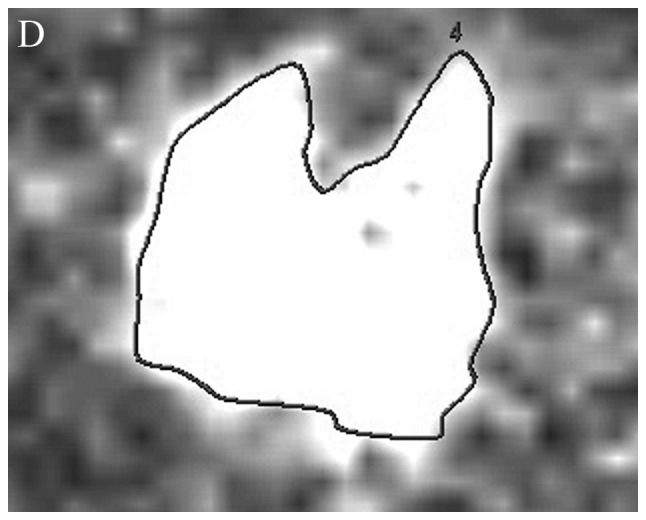

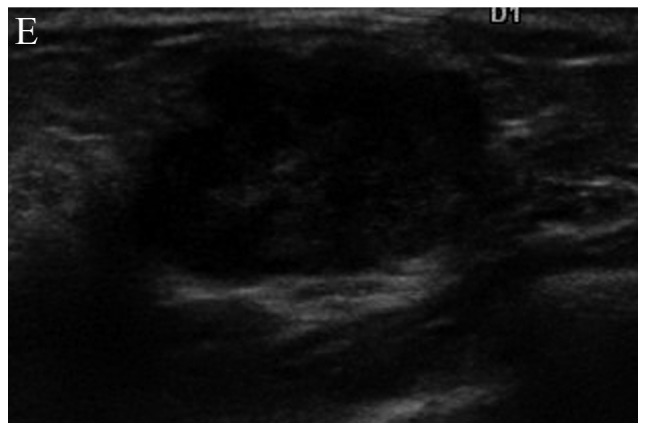

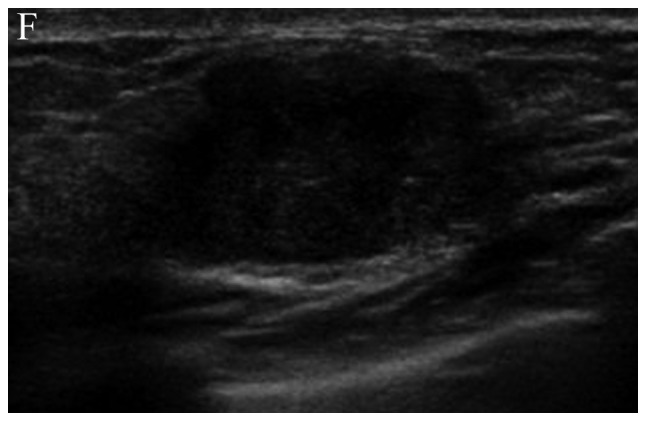
A 40-year-old woman with breast cancer. The breast lesion was enhanced on dynamic MRI before NACT (A) and after final administration of NACT (B). (A and B) The tumor response rate was calculated as 40% by dynamic MRI. A radiologist traced the lesion margin to place the region of interest and calculated the ADC before NACT (C) and after the first cycle of NACT (D). The initial ADC value was 0.879×10^−3^ mm^2^/s, and the %ADC was 14.8% after the first cycle of NACT. Gray-scale ultrasound depicts a hypo-echoic lesion with irregular margins before NACT (E) and after the first cycle of NACT (F). The response rate after the first NACT administration was calculated as −9.5%. NACT, neoadjuvant chemotherapy; ADC, apparent diffusion coefficient.

**Figure 2:**
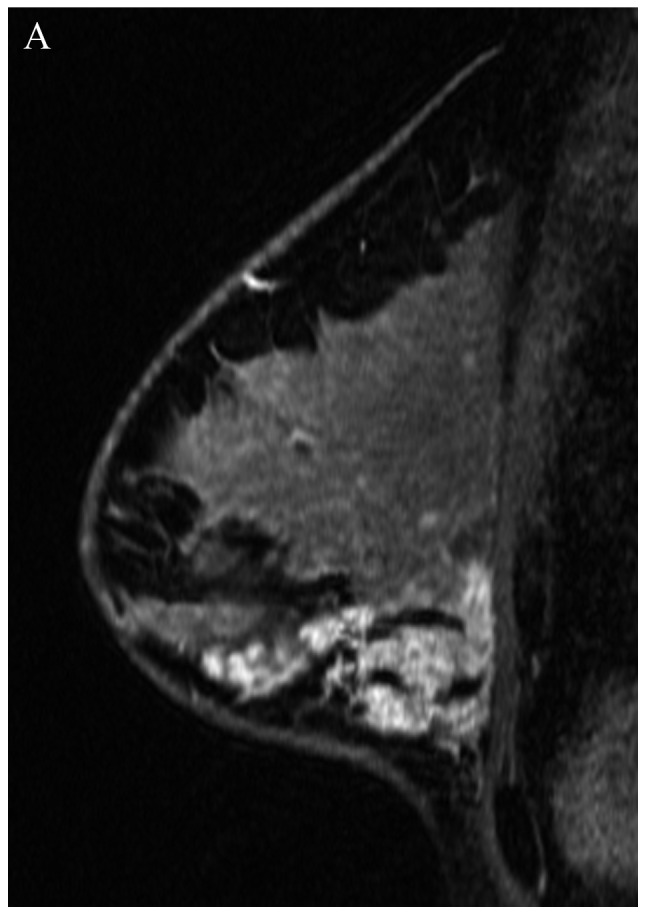

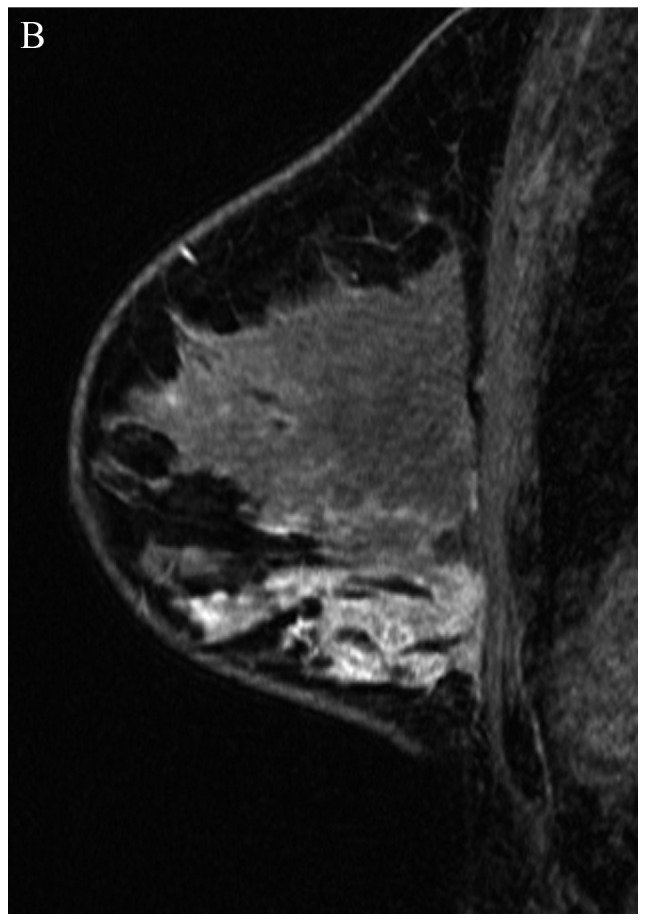

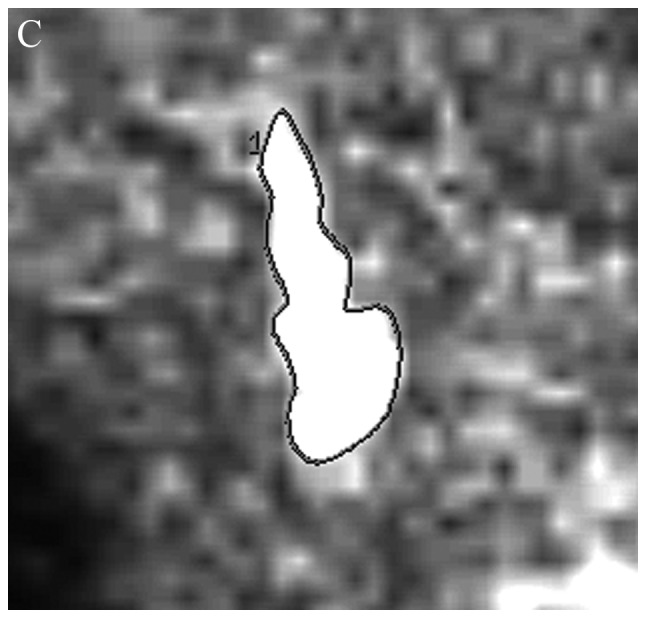

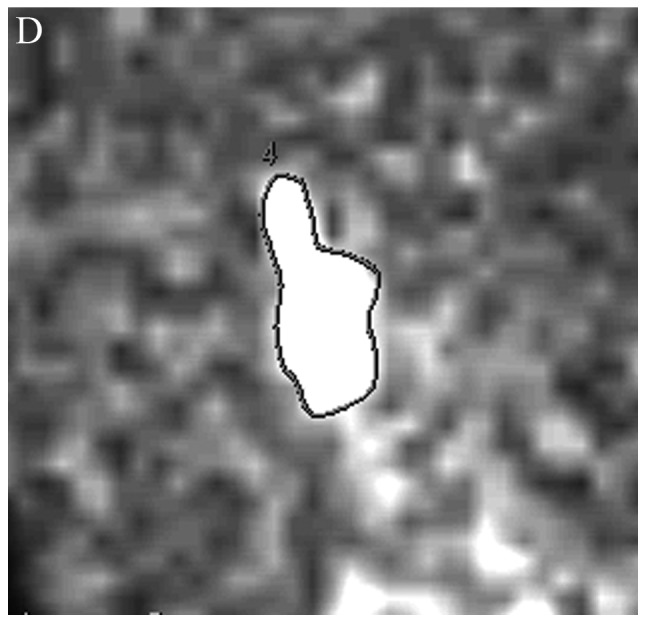

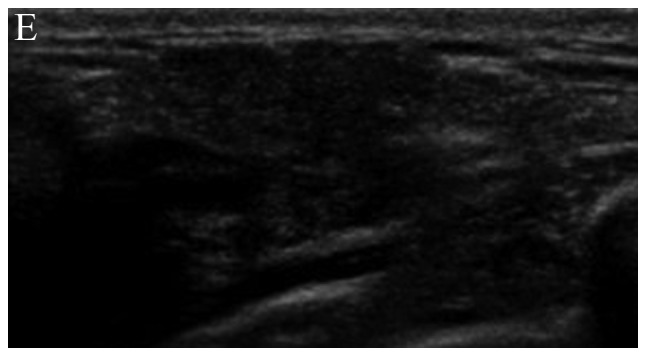

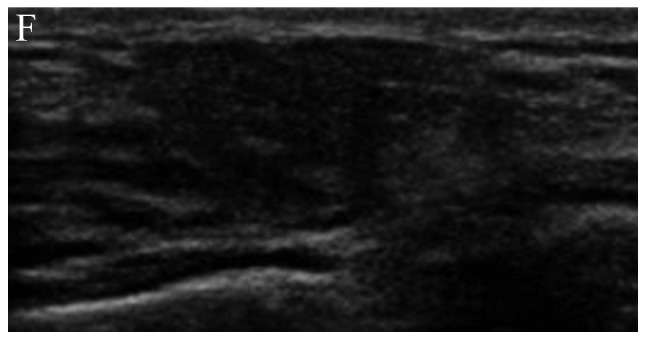
A 41-year-old woman with breast cancer. Dynamic MRI revealed an enhanced lesion that was similar in size both before NACT (A) and after the final cycle of NACT (B); the response rate was 8.3%. (C) The baseline ADC value calculated within the tumor margin was 0.789×10^−3^ mm^2^/s. (D) The %ADC after the first of four cycles of NACT was 2.3%. The response rate between before NACT (E) and after first-time NACT administration (F) was calculated as 5.9% on the ultrasound gray-scale image. NACT, neoadjuvant chemotherapy; ADC, apparent diffusion coefficient.

Dynamic enhanced MRI to measure tumor size was obtained 1–2 days before the first NACT cycle and 10–14 days after the final (fourth) NACT cycle. Dynamic MRI using a three-dimensional fast spoiled gradient-echo sequence (VIBRANT, volume imaging for breast imaging; TR 7.0 ms; TE 4.3 ms; flip angle 10°; FOV 36×36 cm; matrix 512×256; slice thickness 3 mm; gapless; NEX 1) was obtained before and 10 times (every 30 sec) after a bolus injection of 0.1 mmol gadolinium-diethylenetriaminepentaacetic acid (Gd-DTPA)/kg by automatic injector at a rate of 3 ml/sec, followed by a 50-ml saline flush. Tumor sizes were measured on delayed enhanced MRI using the image with the maximum tumor diameter and the signal intensity of the tumor relative to the signal intensity of surrounding breast tissue (Figs. 1A,B and 2A,B). Tumor size was calculated as the biaxial diameter product using the maximum and orthogonal diameters on the maximum dimension of each tumor. Tumor response to NACT was calculated as: 100 × [(tumor size before NACT) - (tumor size after NACT)]/(tumor size before NACT) according to the Response Evaluation Criteria In Solid Tumors (19).

### MRI study (DWI)

All patients were examined using a 3.0-T MRI unit (Signa EXCITE HDx; GE Healthcare, Milwaukee, WI, USA) with an 8-channel, breast, phased-array coil. DWI was performed 1–2 days before and 5–7 days after the first of four cycles of the NACT regimen. DWI was obtained in 2–3 min periods using a transverse spin-echo echo-planar sequence (repetition time, 4,000 ms; echo time 107.3 ms; matrix size, 128×128; section thickness, 4.5 mm; interslice gapless; four signals acquired; field of view, 400 mm). DWI and ADC maps were acquired using b-values of 0 and 1,500 mm^2^/s applied in all directions. Quantitative ADC maps were calculated using commercially available software and an imaging workstation (FuncTool and AW 4.3; GE Healthcare). Regions of interest (ROIs) fitted to the lesion shape were placed on breast cancer lesions on the monitor of the FuncTool workstation to calculate ADC (Figs. 1C,D and 2C,D), based on the following formula:

ADC=[1n (s0/s1)]/(b1-b0)

where 1n is the natural log, b0 = 0 mm^2^/s, b1 = 1,500 mm^2^/s and s0 and s1 are the signal intensities of the lesion on images obtained at each b-value. Changes in the ADC value 5–7 days after the first NACT cycle were determined by calculating the percent change in ADC (%ADC) from baseline (before NACT), with each patient serving as her own control (Figs. 1C,D and 2C,D). The %ADC from before to after the first NACT regimen was calculated based on the following formula:

$$\%\text{ADC}=100\times ({\text{ADC}}^{\text{a}}-{\text{ADC}}^{\text{b}})/{\text{ADC}}^{\text{b}}$$

where ADC^b^ is the ADC of the breast cancer before the first NACT regimen and ADC^a^ is the ADC of the breast cancer after the first NACT regimen.

### Ultrasound study

Each breast mass was scanned using an ultrasound unit (HI VISION Preirus; Hitachi Aloka Medical, Tokyo, Japan) with a 5- to 13-MHz linear-array transducer. An ultrasound study was performed 1–2 days before and 5–7 days after the first of four cycles of the NACT regimen. Tumor size was measured on the gray-scale ultrasound image (Figs. 1E,F and 2E,F). Tumor response to the first of four cycles of NACT was calculated as [%ϕ (US-1)]: 100 × (tumor size before NACT - tumor size after first NACT)/(tumor size before NACT) according to the Response Evaluation Criteria In Solid Tumors (19).

DWI and tumor size on dynamic MRI and on ultrasound were evaluated by three radiologists, K.K., M.N. and N.H., who were blinded to other clinical information and have >15 years’ experience in breast imaging.

### Statistical analysis

Statistical analysis was performed using SPSS version 10.0 software (SPSS Inc., Chicago, IL, USA). Pearson’s correlation test was used to measure the linear association between the tumor response rate and the pre-NACT ADC value [ADC(0)], %ADC and pre-NACT maximum tumor diameter measured by MRI [*ϕ* (MRI-0] and %*ϕ* (US-1). Receiver operating characteristic (ROC) analysis was performed to calculate the area under the curve (AUC) to differentiate responders and non-responders by the independent variable with a significant correlation with the dependent variable (response rate) determined by Pearson’s correlation test. Two-sided tests were used, with values of p<0.05 indicating statistically significant differences.

## Results

**Figure 3:**
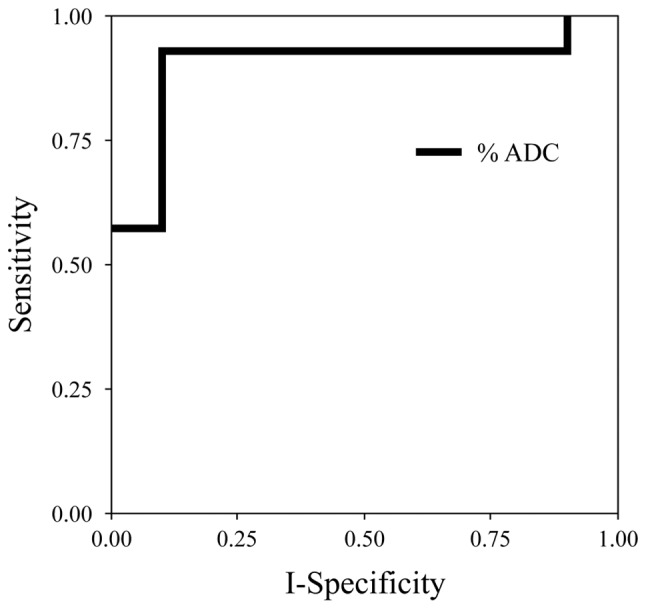
Results of receiver operating characteristics analyses for differentiation of responders and non-responders with %ADC (change in ADC value between before and after first-time neoadjuvant chemotherapy administration). ADC, apparent diffusion coefficient.

Patient characteristics and radiological findings are summarized in Table I. Pearson’s correlation test showed a significant correlation between %ADC and the response rate (r=0.597, p=0.016); none of the other three independent variables were correlated with the response rate (Table II). Therefore, only %ADC was evaluated by ROC analysis (Fig. 3). The AUC of %ADC to differentiate between responders and non-responders on ROC analysis was 0.90 [95% confidence interval, 0.760–1.040]. Breast cancer lesions with high %ADC values responded to NACT (Fig. 1), while those with low %ADC values did not (Fig. 2).

## Discussion

The early prediction of the effectiveness of NACT has the potential to contribute to breast cancer patient prognosis and cosmetic outcome by facilitating the early alteration of the chemotherapy regimen. Among the independent factors extracted from MRI and ultrasound examinations in the present study, only an early change in the ADC after the first cycle of NACT correlated with the tumor response rate and had a sufficient AUC on ROC analysis to differentiate between responders and non-responders. Measurement of ADC by DWI has been reported to be useful to differentiate lesions and evaluate therapeutic efficacy in the breast and other organs. The ADCs of hepatic benign lesions were significantly greater than those of malignant lesions (9). A significant increase in ADC was observed in metastatic lesions that responded to chemotherapy (10). We previously reported that ADC from DWI may evaluate the efficacy of transcatheter arterial chemoembolization for hepatocellular carcinoma as effectively as iodized-oil CT to help in deciding whether to repeat transcatheter arterial chemoembolization (11). The percentage ADC increase was higher in responders than in non-responders after final NACT administration to breast cancer patients (13–17). We concluded that the ADC value from DWI was potentially useful in assessing the response to NACT for breast cancer (14). After final NACT administration, however, patients suffer from the side-effects of large doses of anticancer agents. The fact that time has passed also entails the latent risk that the cancer may have spread throughout the body. The final response must therefore be predicted at an early a point as possible before the start of NACT or after 1 or 2 cycles. Although the pretreatment ADC value was found to be the significant parameter in predicting the response of breast cancer to NACT (15,17), it was not possible to reproduce this finding in the present study. Richard *et al* reported that pretreatment ADC can predict the response of breast cancer to NACT if tumor phenotype is considered (18). Ideally, it should be possible to evaluate the effect of NACT before the start of treatment. An attempt to perform a large-scale study taking into account breast tumor phenotypes would be of value. Pickles *et al* reported that an increase in the mean ADC was noted as early as the first cycle time point of NACT for breast cancer, but a reduction in the mean longest tumor diameter was only noted at the second cycle time point (12). This was a small study involving only 10 subjects, but they found that DWI may provide a suitable biomarker capable of providing an indication of response to treatment prior to tumor size measurement (12). In the present study, which included a larger number of patients, it was possible to demonstrate statistically the advantage reported by Pickles *et al*. Many investigators have verified that both gray-scale ultrasound and Doppler sonography have a high ability to differentiate benign and malignant breast lesions (20–22). Ultrasonography has also been reported to be useful in evaluating axillary lymph node metastases, intraductal cancer spread and outcomes of various conservative therapies for breast cancer (23–28). There have also been attempts to use nuclear medical imaging, magnetic spectroscopy, and contrast-enhanced MRI to evaluate glucose metabolism, cell membrane phospholipid metabolism and enhancement features (16,17,29–31). Although DWI-MRI costs more than ultrasonography, it does not require expensive radiological agents or contrast agents. Magnetic spectroscopy is comparatively expensive, requiring a high-specification MRI device, and it must be used in conjunction with contrast-enhanced MRI to detect breast cancer lesions. The fact that the value of DWI-MRI, which provides comparatively greater versatility among the various diagnostic imaging techniques, for predicting early response to NACT has been demonstrated is highly significant. Anthracycline-based regimens, taxane-based regimens, and third-line regimens have been developed as systemic chemotherapy for breast cancer (1–8,28). Human epidermal growth factor receptor 2 (HER2)-directed therapy for HER2-positive breast cancer and endocrine therapy for hormonal receptor-positive breast cancer are other options (28). In the present study, DWI-MRI successfully predicted the early response to an anthracycline-based regimen. Whether breast cancer determined by DWI-MRI to be unresponsive to an anthracycline-based regimen should be treated with a different systemic therapy or by surgical excision is a question for further study.

In conclusion, change in the ADC after the first cycle of NACT correlated well with the tumor response rate of breast cancer. Calculation of ADC by DWI-MRI was useful in discriminating responders from non-responders to anthracycline-based regimen chemotherapy for breast cancer. Further investigations are required to confirm the benefit of early alteration of systemic therapy based on DWI-MRI response prediction.

## Figures and Tables

**Table I:** Clinical manifestations and imaging findings of the cases examined.

Variables	Values
Age (years)	
Mean	54.3
Range	32–69
TNM	
I	2
II A	13
II B	6
III A	1
III B	1
III C	0
IV	1
ADC (0) (x10^−3^ mm^2^/s)	
Mean	1.006
Range	0.664–1.359
%ADC (%)	
Mean	7.79
Range	−33.8 – +24.13
*ϕ* (MRI-0) (mm)	
Mean	29.8
Range	13–58
%*ϕ* (US-1) (%)	
Mean	8.1
Range	−16.7 – +35.1
Response rate (%)	
Mean	34.1 (14 responders, 10 non-responders)
Range	0–100

TNM, tumor-node-metastasis classification; ADC, apparent diffusion coefficient; ADC (0), ADC value before neoadjuvant chemotherapy (NACT); %ADC, change in ADC value between before and after first-time NACT administration; *ϕ* (MRI-0), maximum tumor diameter measured on MRI before NACT; %*ϕ* (US-1), response rate measured on gray-scale ultrasound image between before and after first-time NACT administration.

**Table II:** Correlation of the response rate (RECIST) by Pearson’s correlation test.

	r	p-value
ADC (0)	−0.272	0.20
%ADC	+0.597	0.016
*ϕ* (MRI-0)	+0.222	0.301
%*ϕ* (US-1)	+0.362	0.083

RECIST, Response Evaluation Criteria In Solid Tumors; r, coefficient of correlation; ADC, apparent diffusion coefficient; ADC (0), ADC value before neoadjuvant chemotherapy (NACT); %ADC, change in ADC value between before and after first-time NACT administration; *ϕ* (MRI-0), maximum tumor diameter measured on MRI before NACT; %*ϕ* (US-1), response rate measured on gray-scale ultrasound image between before and after first-time NACT administration.
